# Novel insights and clinical perception of the patients with acute leukemia transferred to ICU: a multi-center retrospective study spanning 10 years

**DOI:** 10.3389/fmed.2025.1657610

**Published:** 2025-10-27

**Authors:** Qicai Guo, Yanquan Liu, Xiaojun Chen, Jianzhen Shen, Zuotao Li, Minjuan Zeng, Yue Yin, Jiachen Xie, Ye Li, Huidong Guo, Zuohong Cao

**Affiliations:** ^1^Department of Emergency, The First Affiliated Hospital of Gannan Medical University, Ganzhou, Jiangxi, China; ^2^Jiangxi Health Commission Key Laboratory of Leukemia, Department of Hematology, The Affiliated Ganzhou Hospital (Ganzhou People’s Hospital) of Nanchang University, Ganzhou, Jiangxi, China; ^3^Department of Hematology and Rheumatology, The Affiliated Hospital of Putian University, Putian, Fujian, China; ^4^National Key Laboratory of Hematology, Fujian Institute of Hematology, Fujian Medical University Union Hospital, Fuzhou, Fujian, China; ^5^Department of Intensive Medicine (Comprehensive ICU), The First Affiliated Hospital of Gannan Medical University, Ganzhou, Jiangxi, China; ^6^The Affiliated Hospital (First School of Clinical Medicine) of Guangdong Medical University, Zhanjiang, Guangdong, China; ^7^Department of Pathology, The First Affiliated Hospital of Gannan Medical University, Ganzhou, Jiangxi, China

**Keywords:** acute leukemia, hematological malignancy, ICU, prognosis, rescue treatment, critical care medicine, clinical early warning

## Abstract

**Background:**

Intensive care unit (ICU) is a professional and special ward for the treatment of various critical and severe diseases in clinical medical institutions, which is an important embodiment of the strength of critical and severe treatment in a hospital. Acute leukemia (AL) is one of the most common hematological malignancies with the most serious condition and poor prognosis. Different from other hematological malignancies, AL kills many young patients every year, although the treatment for AL has been perfected and put into clinical treatment for many years, however, due to the severity and rapid progression of AL, it is still necessary to strengthen the ability of nursing and medical treatment. Careful analysis and discussion of the rescue treatment and nursing of AL patients transferred to ICU is particularly necessary to improve the rescue ability of malignant hematological tumors in the critical stage and improve the rescue success rate of AL.

**Objective:**

The purpose of this paper is to discuss and analyze the clinical features, diagnosis, treatment and prognosis of AL patients transferred to ICU, so as to provide suggestions for improving the ability to treat malignant hematological tumors in critical stage and improve the rescue measures for AL. More importantly, our aim was to provide evidence-based insights for early warning systems and multidisciplinary approaches in managing critically ill patients with hematological malignancies, investigate the clinical characteristics and prognostic factors of AL patients requiring ICU admission, providing valuable insights for improving diagnosis, nursing, prognosis assessment, and palliative care in hematology and critical care medicine.

**Methods:**

Clinical data were retrospectively collected and systematically organized for AL patients transferred to the ICU from the First Affiliated Hospital of Gannan Medical University, Fujian Medical University Union Hospital, the Affiliated Hospital of Putian University, and the Affiliated Hospital of Guangdong Medical University during January 2014 to January 2025. The collected data included general patient characteristics, age at onset, treatment regimens, routine hematological indicators, cytogenetic and molecular biological abnormalities, extramedullary organ infiltration, and acute physiology and chronic health evaluation scores (APACHE II score), ICU duration and outcomes, reasons for ICU admission, treatment courses during ICU stays, and relevant laboratory and imaging findings. This study aimed to analyze and discuss the clinical features, diagnostic and therapeutic approaches, and prognosis of leukemia patients admitted to the ICU.

**Results:**

A total of 357 AL patients, aged 16.5 ~ 77 years, were included in this study, comprising 216 males and 141 females. The time interval from AL diagnosis to ICU admission ranged from 0.03 to 144 months, with a median of 1 month. The length of ICU stay varied between 1 and 30 days. From the perspective of the unique molecular biology and cytogenetics of AL, we found FLTS-ITD was independent risk factors for mortality of AML, while E2A-PBX1 and DNMT3A were independent risk factors for mortality of ALL. Regardless of whether the subtype of AL patients included in this study was AML or ALL, patients with complex karyotypes accounted for the largest proportion. Meanwhile, age, leukemia type, heart failure, APACHE II score, WBC, PLT, LDH, PCT, APTT significantly affected the time from diagnosis to transfer to the ICU in AL patients (*p* < 0.05), accompanied by the gene mutation of WT1, FLT3-ITD, and TP53 significantly affected the time from diagnosis to transfer to the ICU in AML patients (*p* < 0.05), and FLT3-ITD, E2A-PBX1, DNMT3A, HOX11, RUNX1 significantly affected the time from diagnosis to transfer to the ICU in ALL patients (*p* < 0.05). Univariate analysis revealed that heart failure, sepsis, continuous renal replacement therapy (CRRT), administration of two or more treatments simultaneously, APACHE II score ≥20, and procalcitonin (PCT) levels were significantly associated with prognosis. Multivariate analysis indicated that heart failure, CRRT, and APACHE II score ≥20 were independent risk factors for mortality. COX univariate analysis suggested that heart failure, vasopressor use, and APACHE II score were influencing factors for overall survival (OS), while multivariate analysis confirmed that vasopressor use was an independent risk factor for OS.

**Conclusion:**

The prognosis and outcomes for AL patients transferred to the ICU were generally poor. Some molecular biological and cytogenetic indicators can be used as early warning indicators for AL patients’ transfer to the ICU or short-term death. Acute respiratory failure, sepsis, and severe infections were the primary reasons for ICU admission. Heart failure, CRRT, and APACHE II score ≥20 were identified as independent risk factors for mortality, while vasopressor use was an independent risk factor for OS.

## 1 Introduction

Acute leukemia (AL) is a hematological malignancy characterized by the aberrant proliferation of primitive or immature cells in the bone marrow and peripheral blood, and it is marked by rapid onset, high clinical severity, and complex complications (1–3). Despite decades of innovation in hematology, including advancements in chemotherapy combined with bone marrow transplantation, targeted therapies, immunotherapies, and the use of epigenetic drugs, clinical benefits remain limited to certain subgroups of AL patients. The complexity of AL pathogenesis varies significantly across its numerous subtypes, leading to short-term recurrence and mortality in many cases (4, 5). Clinically, treatment-related complications such as severe infections, multiple organ failure, and tumor lysis syndrome remain the primary reasons for admission to the Intensive Care Unit (ICU), and the in-ICU mortality rate for these patients could reach 40–60%, significantly higher than that of patients in general hematology wards (6–8). Correspondingly, AL often presents as an acute and life-threatening condition, leaving clinicians with limited time for intervention. Besides, AL remains a major cause of morbidity and mortality among young individuals, posing a significant threat to public health and social stability (9–11). Consequently, identifying risk factors, prognostic characteristics, and intervention strategies for acute leukemia patients transferred to the ICU is crucial for optimizing clinical decision-making and reducing mortality.

This study retrospectively analyzed the diagnostic and therapeutic data of AL patients admitted to the ICU at four medical centers over the past decade: the First Affiliated Hospital of Gannan Medical University, Fujian Medical University Union Hospital, the Affiliated Hospital of Putian University, and the Affiliated Hospital of Guangdong Medical University. Our aim was to provide evidence-based insights for early warning systems and multidisciplinary approaches in managing critically ill patients with hematological malignancies, investigate the clinical characteristics and prognostic factors of AL patients requiring ICU admission, providing valuable insights for improving diagnosis, nursing, prognosis assessment, and palliative care in hematology and critical care medicine.

## 2 Methods and materials

### 2.1 Research object

A retrospective analysis was conducted on 357 cases of AL patients admitted to the ICU of the First Affiliated Hospital of Gannan Medical University, Fujian Medical University Union Hospital, the Affiliated Hospital of Putian University, and the First Affiliated Hospital of Guangdong Medical University between January 2014 and January 2025. Among these patients, 216 were male and 141 were female. There were 204 cases of acute myeloid leukemia (AML) and 153 cases of acute lymphoblastic leukemia (ALL). Patient ages ranged from 16.5 to 77 years, with a median age of 42.15 years. All patients were classified according to clinical manifestations, complete blood count, and the MICM international typing criteria, which include bone marrow cell morphology (including cytochemical staining), immunophenotyping, cytogenetics, and molecular biology. Ethical approval was obtained from the medical ethics committees of all participating centers, and informed consent was provided by all patients or their families. For patients with multiple ICU admissions, only data from their first ICU stay were included in the analysis.

### 2.2 Diagnostic method

All diagnosed AL patients included in this study underwent bone marrow aspiration biopsy upon initial presentation. Bone marrow smears were stained using morphological and cytochemical techniques, while bone marrow biopsies were stained using immunohistochemistry. Corresponding bone marrow samples were collected for further analysis, including flow cytometric immunophenotyping, chromosomal analysis, and molecular genetic testing (e.g., fusion and mutant genes). A comprehensive diagnosis of AL was made based on the results of these tests.

### 2.3 Research criteria

The inclusion criteria of this clinical study were: 1. ≥16 years of age, standard treatment plan for admission, complete clinical data and course data (including ICU period). 2. Clinical manifestations consistent with leukemia. 3. Newly diagnosed AL after admission, with no prior clinical interventions at other hospitals. 4. Diagnosis confirmed using peripheral blood smear, bone marrow routine examination (including cytochemical staining), bone marrow pathological biopsy, flow cytometric immunophenotyping, cytogenetic and molecular biological detection.

Exclusion Criteria: 1. Patients lost to follow-up. 2. Patients preliminarily diagnosed with AL based solely on peripheral blood smear or bone marrow biopsy without hospital admission. 3. Patients with a confirmed diagnosis of AL who did not receive internationally recognized standard treatments. 4. Patients with a history of other malignancies (complex or multiple cancers). 5. Patients with incomplete medical records who abandoned ICU-specific diagnosis, treatment, and intensive care interventions. 6. Patients who declined participation due to privacy concerns. 7. Patients do not agree to be included in this study due to mental or psychological factors.

### 2.4 Study implementation process

**Figure 1:**
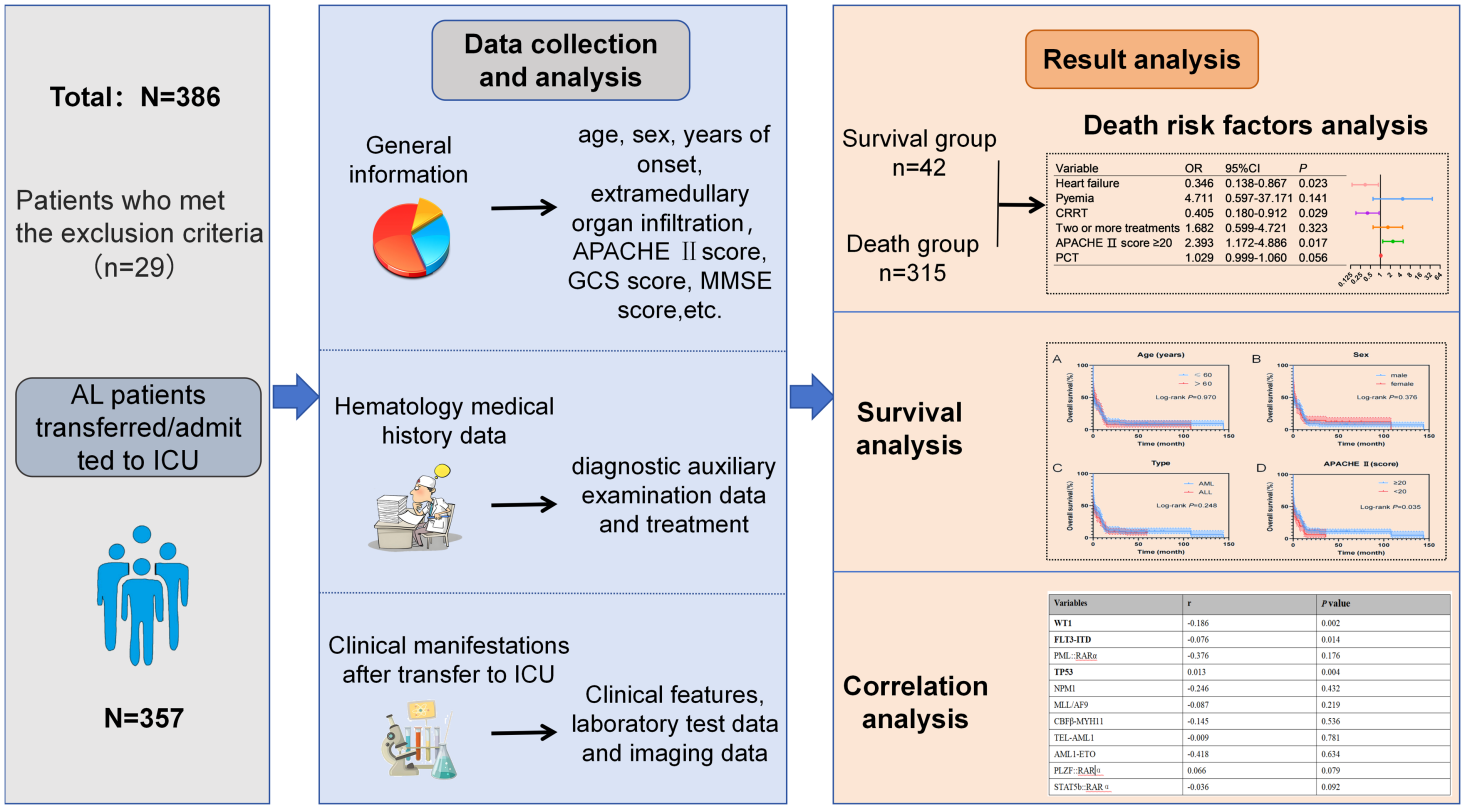
The technical roadmap of this research (AL: acute leukemia; APACHE II score: acute physiology and chronic health status score).

The clinical data collected included demographic information, previous hematological diagnoses and treatments, hematological parameters, bone marrow findings, flow cytometric immunophenotyping, cytogenetic and molecular biological results, extramedullary organ infiltration, acute physiology and chronic health evaluation score (APACHE II score), Glasgow coma scale (GCS score), mini-mental state examination (MMSE score), ICU admission duration and outcomes, reasons for ICU admission, ICU treatment modalities (e.g., invasive mechanical ventilation, hormone therapy, blood transfusion components, immunoglobulins, vasoactive drugs), and relevant laboratory and imaging findings. Hospital electronic medical record systems were used to retrieve patient information regarding hospitalization, diagnosis, and prognosis. Follow-up was conducted via telephone or email with patients and their families. The specific research implementation process and technical roadmap are illustrated in Figure 1.

### 2.5 Efficacy evaluation and follow-up

Efficacy evaluation followed the latest WHO guidelines for AL diagnosis and treatment (12–17), available through NCBI. Follow-up involved reviewing inpatient diagnosis, treatment, and prognosis information via the hospital electronic medical record system and contacting patients and their families by telephone or email. Follow-up concluded on March 1, 2025, with the last follow-up date serving as the endpoint for patients lost to follow-up. The median follow-up time was 1 (0.03–144) month. Overall survival (OS) was defined as the time from diagnosis to death or the last follow-up for AL patients included in this study.

### 2.6 Statistical analysis

Statistical analyses were performed using IBM SPSS 26.0 software. Normally distributed measurement data were expressed as *Mean* ± *SD* and analyzed using *t*-tests. Non-normally distributed measurement data were expressed as median (interquartile range) and analyzed using Mann–Whitney tests. Count data were expressed as *n* (%) and analyzed using chi-square or Fisher’s exact tests. Binary logistic regression was used for univariate and multivariate analyses of mortality risk factors. Kaplan–Meier curves and Log-rank tests were used for survival analysis, while Cox proportional hazards models were applied for univariate and multivariate analyses of OS risk factors. Survival curves were generated using GraphPad Prism 9.0 software, and other clinical data were analyzed descriptively. Statistical significance was set at *p* < 0.05.

## 3 Results

### 3.1 General clinical data of AL patients in ICU

This study included 357 AL patients aged 16.5 ~ 77 years, comprising 216 males and 141 females. All patients met the diagnostic criteria outlined in the WHO guidelines for AL. The interval from AL diagnosis to ICU admission ranged from 0.03 to 144 months, with a median of 1 month. ICU stays lasted 1 ~ 30 days. Treatments administered after ICU admission included vasoactive drugs (*n* = 56), hormone shock therapy (*n* = 76), mechanical ventilation (*n* = 75), plasma exchange (*n* = 6), continuous renal replacement therapy (CRRT, *n* = 42), and combinations of two or more treatments (*n* = 102). Severe infections were common complications, affecting 273 patients with lung infections, 135 with sepsis, 6 with intracranial infections, 9 with oral infections, 12 with abdominal infections, and 18 with infections in other tissues or organs. Detailed general information and basic clinical characteristics of all ICU-admitted AL patients are summarized in Table 1.

**Table 1:** Baseline data and clinical features of 357 AL patients transferred to ICU.

Clinical characteristics	Results
Gender [*n* (%)]	
Male	216 (60.50)
Female	141 (39.50)
Age [*n* (%)]	
≤ 60 year-old	243 (68.07)
> 60 year-old	114 (31.93)
Types of AL disease [*n* (%)]	
Acute myeloid leukemia	204 (57.14)
Acute lymphoblastic leukemia	153 (42.86)
Duration of AL disease (month)	5.89 [0.06–8.00]
Total length of stay in ICU (days)	4.13 [1.00–5.00]
WBC (×10^9^/L)	104.81 [0.93–88.70]
HGB (g/L)	20.59 [45.00–68.00]
PLT (×10^9^/L)	34.29 [5.00–44.00]
LDH (U/L)	2146.48 [383.00–2125.00]
PCT (ng/mL)	15.24 [0.69–10.80]
IL-6 (ng/mL)	1618.76 [23.81–242.24]
Abnormal blood biochemical index [*n* (%)]	
Abnormal liver function	234 (65.55)
Abnormal renal function	296 (82.91)
Abnormal coagulation function	229 (64.15)
Electrolyte disturbance	303 (84.87)
Bleeding in organ system [*n* (%)]	
Dermatorrhagia	108 (30.25)
Gastrointestinal hemorrhage	78 (21.85)
Cerebral hemorrhage	72 (20.17)
Urinary hemorrhage	25 (7.00)
Complicated with thrombus [*n* (%)]	11 (3.08)
Combined infection [*n* (%)]	
Pulmonary infection	273 (76.47)
Septicemia	135 (37.82)
Intracranial infection	6 (1.68)
Oral infection	9 (2.52)
Abdominal infection	12 (3.36)
Infection of tissues and organs	18 (5.04)
APACHE II score [*n* (%)]	
0~10 scores	25 (7.00)
11~15 scores	42 (11.76)
15~20 scores	98 (27.45)
≥ 20 scores	192 (53.78)
GCS score [*n* (%)]	
< 3 scores	14 (3.92)
3~8 scores	37 (10.37)
9~12 scores	208 (58.26)
13~15 scores	98 (27.45)
MMSE score [n (%)]	
0~9 scores	11 (3.08)
10~20 scores	29 (8.12)
21~26 scores	174 (48.74)
27~30 scores	143 (40.06)

### 3.2 Analysis of reasons for AL patients transferred to ICU

**Figure 2:**
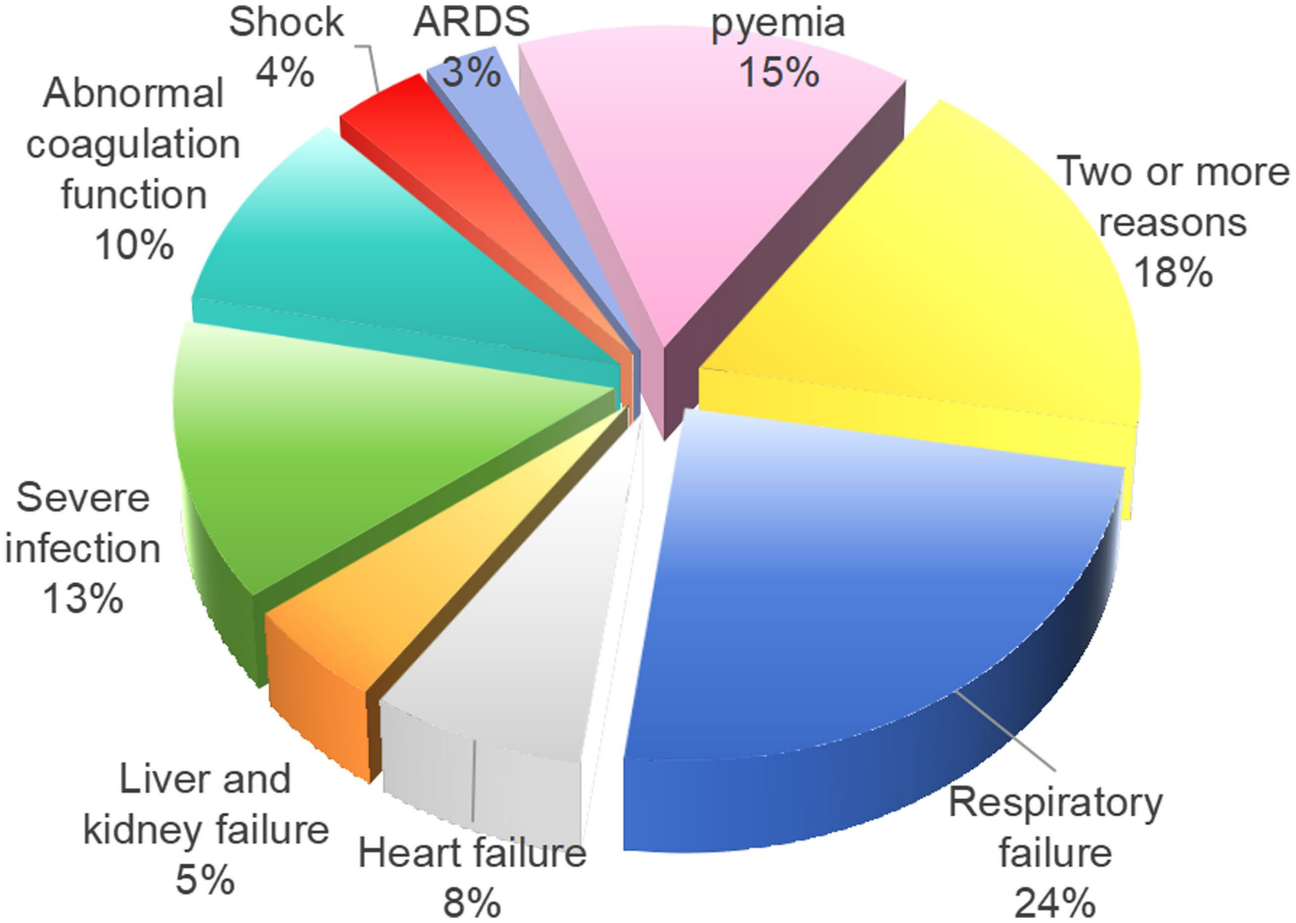
Proportion of 357 AL patients transferred to the ICU for different reasons.

As presented in Table 2, among the 357 cases of AL patients included in this study, 87 cases were admitted to the ICU due to “acute respiratory failure,” 27 cases due to “acute heart failure,” 18 cases due to “liver and kidney failure,” 48 cases due to “severe infection,” 35 cases due to “clinically uncontrollable coagulation dysfunction,” 13 cases due to “shock,” 10 cases due to “acute respiratory distress syndrome (ARDS),” and 54 cases due to “sepsis.” Notably, 39 cases patients required readmission to the ICU, and 65 cases of patients were admitted for two or more reasons listed above. The detailed proportions of AL patients transferred to the ICU for various reasons are illustrated in Figure 2.

**Table 2:** Statistics of 357 cases of AL patients transferred to ICU.

Reasons for AL patients transferred to ICU	Cases [*n* (%)]
Acute respiratory failure	87 (24.37)
Acute heart failure	27 (7.56)
Liver and kidney failure	18 (5.04)
Severe infection	48 (13.45)
Clinically uncontrollable coagulation dysfunction	35 (9.80)
Shock	13 (3.64)
Acute respiratory distress syndrome (ARDS)	10 (2.80)
Sepsis	54 (15.13)
Combine two or more reasons	65 (18.21)

### 3.3 Treatment and outcomes of AL patients transferred to ICU

**Figure 3:**
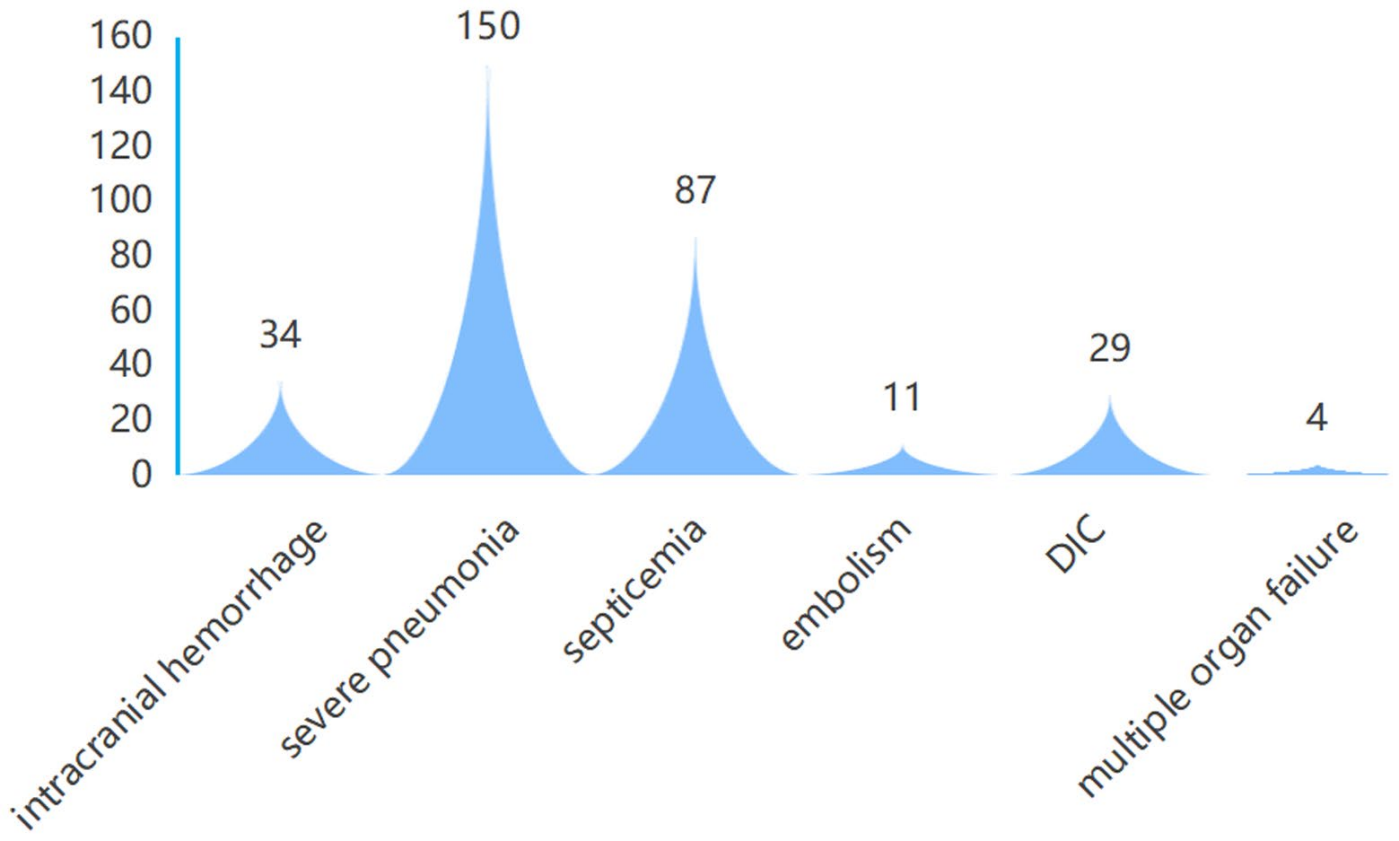
The causes of death among all AL patients transferred to ICU.

Among the all cases of AL patients included in this study, 56 cases received vasoactive drugs, 76 cases underwent hormone shock therapy, 75 cases required mechanical ventilation, 6 cases underwent plasma exchange, 42 cases received CRRT, and 102 cases received two or more treatments simultaneously. Among all patients, 315 cases died after ICU admission (including 45 cases in-hospital deaths and 270 cases post-discharge deaths following family-initiated treatment abandonment), resulting in a case fatality rate of 88.24%. Three patients died within 28 days of ICU admission, and 28 cases remained alive at the one-year follow-up mark. Among the 315 cases deceased patients, causes of death included: 34 cases of intracranial hemorrhage, 150 cases of severe pneumonia, 87 cases of septicemia, 11 cases of embolism, 29 cases of disseminated intravascular coagulation (DIC), and 4 cases of multiple organ failure secondary to differentiation syndrome. The causes of death among all AL patients transferred to ICU included in this study are presented in Figure 3.

### 3.4 Molecular biology detection

**Figure 4:**
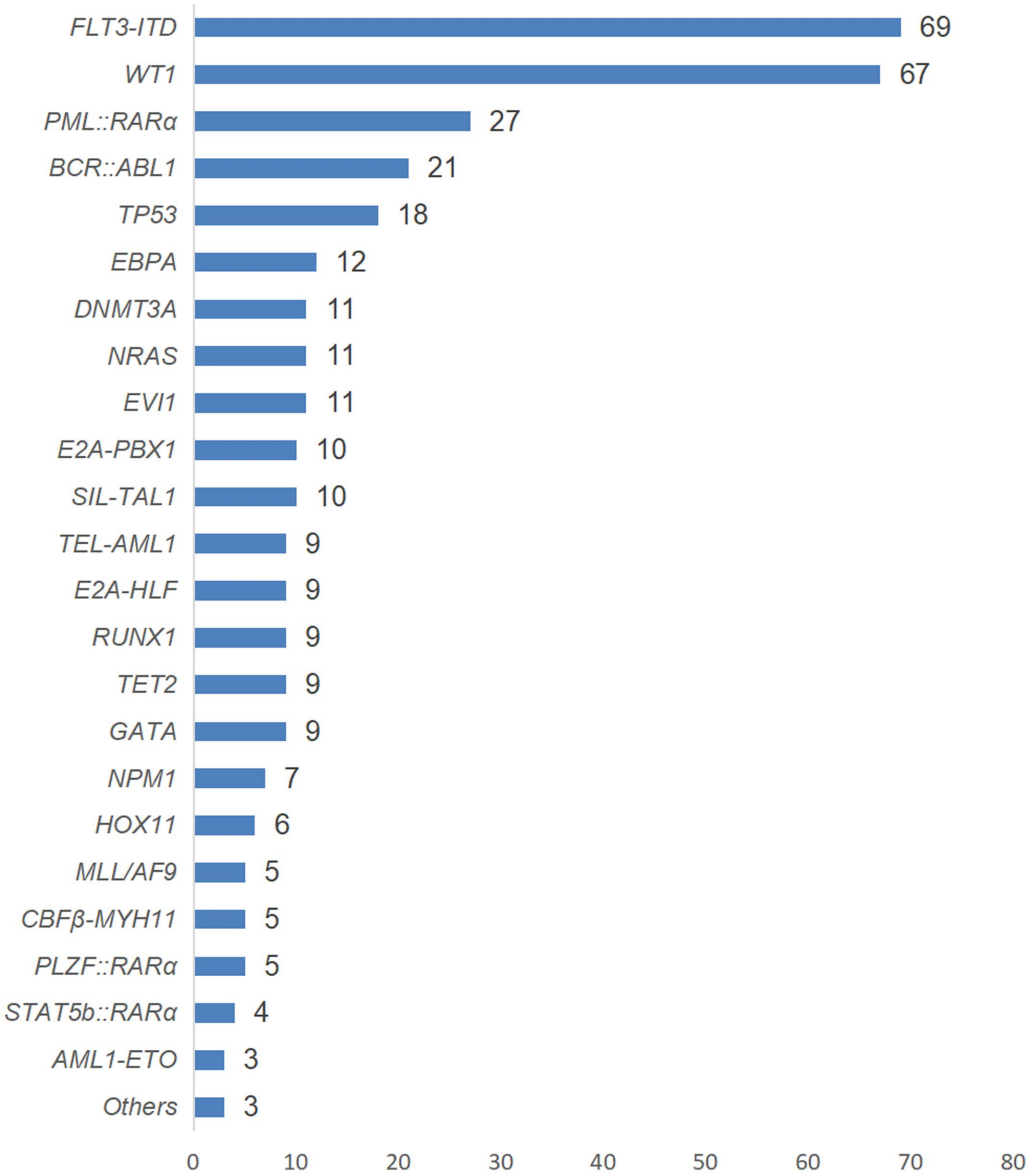
The genetic conditions of AL patients with positive test results in this study.

Among the 357 AL patients included in this study, a total of 352 cases completed molecular biological tests (199 cases of AML and 153 cases of ALL), and 343 cases obtained positive results (194 cases of AML and 149 cases of ALL). The abnormal results of molecular biology are detailed in Figure 4.

### 3.5 Univariate analysis of the prognosis of AL patients transferred to the ICU based on molecular biological results

Univariate analysis is shown in Tables 3, 4, AL patients transferred to the ICU were divided into two subtypes, namely AML and ALL. Subsequently, our statistical analysis revealed that FLT3-ITD, PML-RARα, TEL-AML1, and AML1-ETO were related to the prognosis of AML patients. BCR-ABL1, FLT3-ITD, E2A-PBX1, DNMT3A, NRAS, EVI1, HOX11, RUNX1 and GATA are related to the prognosis of ALL patients.

**Table 3:** Univariate analysis of risk factors for death of genes and AML patients transferred to ICU.

Causes of death and related factors	AMLpatients (*n* = 204)	Outcome during ICU stay		χ^2^	*p* value
		Survivors (*n* = 26)	Non-survivors (*n* = 178)		
WT1	67 (32.84%)	12 (46.15%)	55 (30.90%)	2.394	0.122
FLT3-ITD	56 (27.45%)	15 (57.69%)	41 (23.03%)	13.683	**< 0.001**
PML::RARα	27 (13.24%)	17 (65.38%)	10 (5.62%)	70.567	**< 0.001**
TP53	6 (2.94%)	2 (7.69%)	4 (2.24%)	2.356	0.125
NPM1	7 (3.43%)	2 (7.69%)	5 (2.81%)	1.633	0.201
MLL/AF9	5 (2.45%)	2 (7.69%)	3 (1.68%)	3.424	0.064
CBFβ-MYH11	5 (2.45%)	1 (3.84%)	4 (2.24%)	0.243	0.622
TEL-AML1	9 (4.41%)	4 (15.38%)	5 (2.81%)	8.508	**0.004**
AML1-ETO	3 (1.47%)	2 (7.69%)	1 (0.56%)	7.961	**0.005**
PLZF::RARα	5 (2.45%)	2 (7.69%)	3 (1.68%)	3.424	0.064
STAT5b::RARα	4 (1.96%)	1 (3.84%)	3 (1.68%)	0.551	0.458

The bold values represent *p* values < 0.05, which is statistically significant.

**Table 4:** Univariate analysis of risk factors for death of genes and ALL patients transferred to ICU.

Causes of death and related factors	ALLpatients (*n* = 153)	Outcome during ICU stay		χ^2^	*P* value
		Survivors (*n* = 16)	Non-survivors (*n* = 137)		
BCR::ABL1	22 (14.38%)	7 (43.75%)	15 (10.95%)	12.520	**< 0.001**
FLT3-ITD	13 (8.49%)	7 (43.75%)	6 (4.38%)	28.563	**< 0.001**
E2A-HLF	9 (5.88%)	2 (12.50%)	7 (5.11%)	1.413	0.234
E2A-PBX1	9 (5.88%)	3 (18.75%)	6 (4.38%)	5.344	**0.021**
SIL-TAL1	10 (6.54%)	2 (12.50%)	8 (5.84%)	1.040	0.308
DNMT3A	11 (7.19%)	4 (25.00%)	7 (5.11%)	8.495	**0.004**
EBPA	12 (7.84%)	2 (12.50%)	10 (7.30%)	0.536	0.464
NRAS	11 (7.19%)	5 (31.25%)	6 (4.38%)	15.502	**< 0.001**
TP53	12 (7.84%)	3 (18.75%)	9 (6.57%)	2.941	0.086
EVI1	11 (7.19%)	6 (37.50%)	5 (3.65%)	24.602	**< 0.001**
HOX11	6 (3.92%)	3 (18.75%)	3 (2.19%)	10.428	**0.001**
RUNX1	10 (6.54%)	4 (25.00%)	6 (4.38%)	9.972	**0.002**
TET2	9 (5.88%)	2 (12.50%)	7 (5.11%)	1.413	0.234
GATA	8 (5.23%)	3 (18.75%)	5 (3.65%)	6.592	**0.010**

The bold values represent *p* values < 0.05, which is statistically significant.

### 3.6 Multivariate analysis of the prognosis of AL patients transferred to the ICU based on molecular biological results

**Figure 5:**
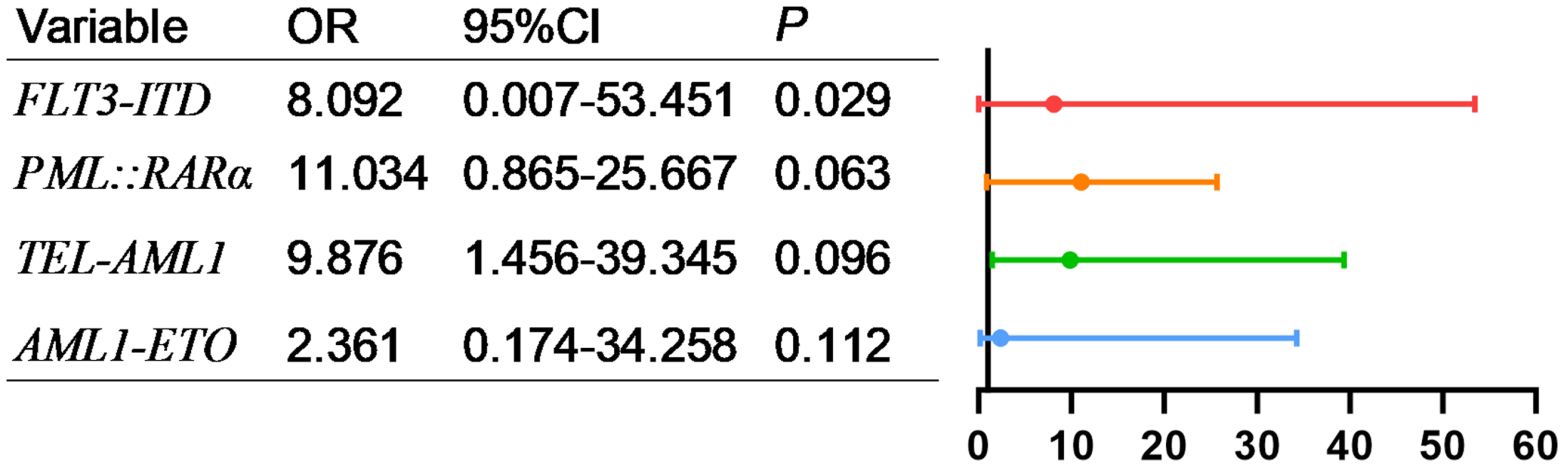
Multivariate logistic regression analysis of risk factors for death of genes and AML patients transferred to ICU.

**Figure 6:**
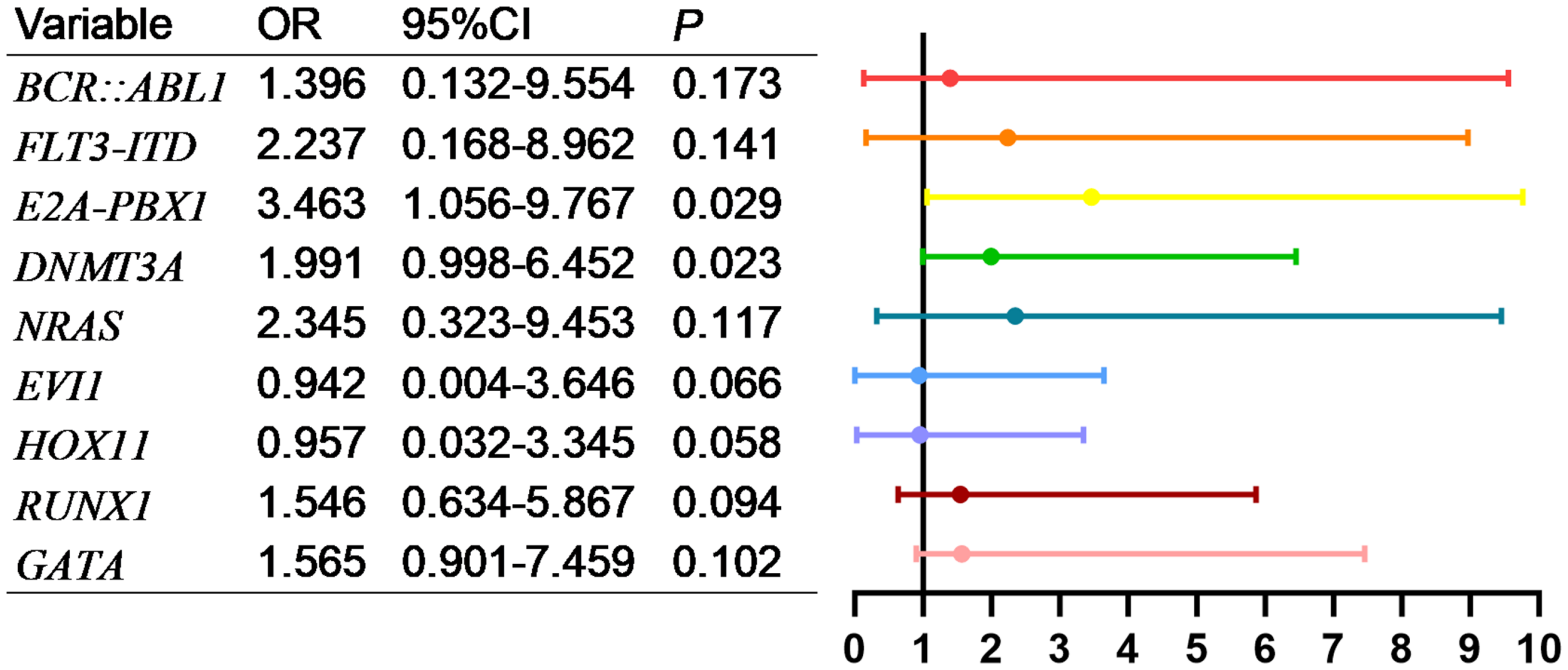
Multivariate logistic regression analysis of risk factors for death of genes and ALL patients transferred to ICU.

**Figure 7:**
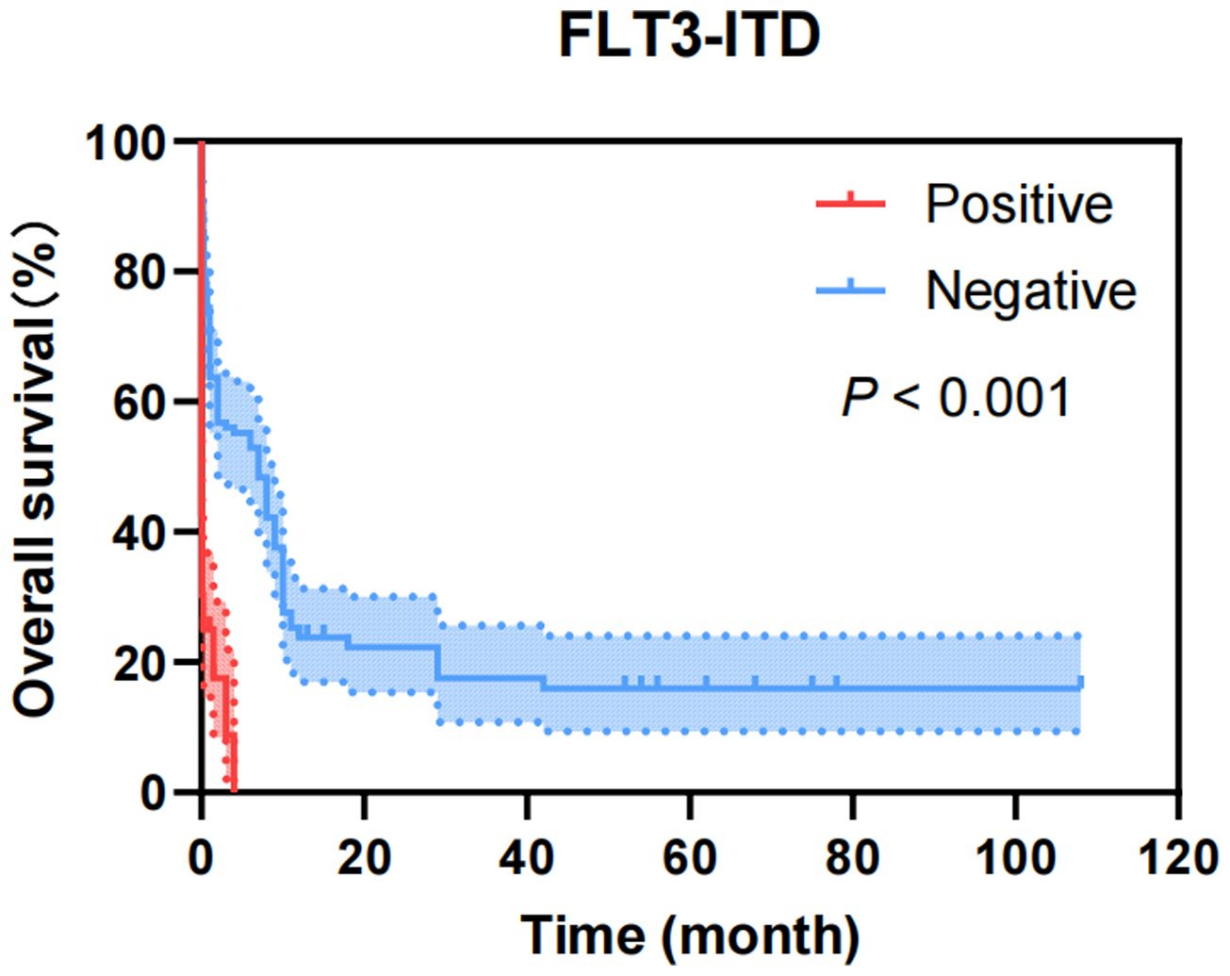
Kaplan–Meier overall survival curves of FLT3-ITD of AML patients transferred to ICU.

**Figure 8:**
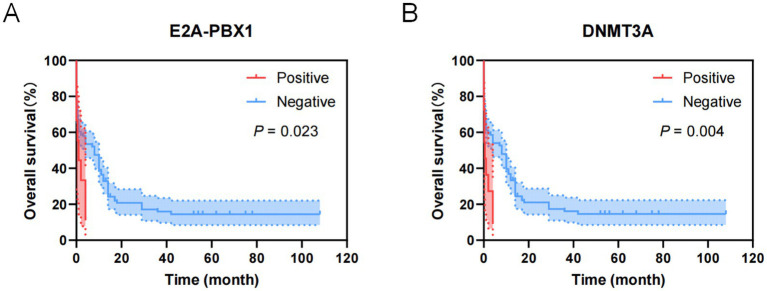
Kaplan–Meier overall survival curves for genes of ALL patients transferred to ICU. **(A)** The overall survival curves of E2A-PBX1 of ALL patients transferred to ICU. **(B)** The overall survival curves of DNMT3A of ALL patients transferred to ICU.

As shown in Figure 5, factors with *p* < 0.05 in the univariate analysis were included in multivariate analysis, revealing that FLT3-ITD were independent risk factors for mortality of AML. What’s more, as shown in Figure 6, factors with *p* < 0.05 in the univariate analysis were included in multivariate analysis, revealing that *E2A-PBX1*, *DNMT3A* were independent risk factors for mortality of ALL. The above genes were, respectively, included in the OS survival analysis of AML patients and ALL patient, and the results showed that FLT3-ITD is the prognostic factor affecting the OS of AML patients, and E2A-PBX1 and DNMT3A are the prognostic factors affecting the OS of ALL patients (Tables 5, 6). Furthermore, as shown in Figure 7, FLT3-ITD is a prognostic factor affecting the OS of AML patients. However, as shown in Figure 8, E2A-PBX1 and DNMT3A are prognostic factors affecting the OS of ALL patients.

**Table 5:** Univariate Cox regression analysis of genes and OS in AML patients transferred to the ICU.

AML			
Clinical features	*n* (%)	χ^2^ value	*P* value
WT1		23.580	0.108
Positive	67		
Negative	132		
FLT3-ITD		78.170	**< 0.001**
Positive	56		
Negative	143		
PML::RARα		23.875	0.102
Positive	27		
Negative	172		
TP53		11.960	0.058
Positive	6		
Negative	193		
NPM1		0.103	0.789
Positive	7		
Negative	192		
MLL/AF9		37.565	0.299
Positive	5		
Negative	194		
CBFβ-MYH11		22.238	0.147
Positive	5		
Negative	194		
TEL-AML1		44.631	0.097
Positive	9		
Negative	190		
AML1-ETO		179.450	0.211
Positive	3		
Negative	196		
PLZF::RARα		29.981	0.188
Positive	5		
Negative	194		
STAT5b::RARα		17.967	0.065
Positive	4		
Negative	195		

The bold values represent *p* values < 0.05, which is statistically significant.

**Table 6:** Univariate Cox regression analysis of genes and OS in ALL patients transferred to the ICU.

ALL			
Clinical features	*n* (%)	χ^2^ value	*P* value
BCR::ABL1		0.053	0.175
Positive	22		
Negative	131		
FLT3-ITD		0.002	0.586
Positive	13		
Negative	140		
E2A-HLF		5.024	0.086
Positive	9		
Negative	144		
E2A-PBX1		5.173	**0.023**
Positive	9		
Negative	144		
SIL-TAL1		1.747	0.117
Positive	10		
Negative	143		
DNMT3A		8.292	**0.004**
Positive	11		
Negative	142		
EBPA		0.998	0.396
Positive	12		
Negative	141		
NRAS		3.564	0.068
Positive	11		
Negative	142		
TP53		4.382	0.056
Positive	12		
Negative	141		
EVI1		0.088	0.123
Positive	11		
Negative	142		
HOX11		0.015	0.447
Positive	6		
Negative	147		
RUNX1		6.364	0.059
Positive	10		
Negative	143		
TET2		1.996	0.457
Positive	9		
Negative	144		
GATA		1.278	0.261
Positive	8		
Negative	145		

The bold values represent *p* values < 0.05, which is statistically significant.

### 3.7 Cytogenetic detection

Among 357 patients, a total of 341 completed cytogenetic detection (195 cases of AML and 146 cases of ALL), and 297 cases showed abnormal results (169 cases of AML and 128 cases of ALL). As shown in Table 7, chromosome karyotype analysis was conducted on 195 AML patients. Among them, 26 cases (13.33%) had normal karyotypes, and among 146 ALL patients, 18 cases (12.33%) had normal karyotypes. Whether it is AML or ALL, patients with complex karyotypes account for the largest proportion. The second most common abnormal chromosomal karyotype among AML patients is t(15; 17)(q24; q21) (9.23%), the second most common abnormal chromosomal karyotype among ALL patients was −20 (17.12%).

**Table 7:** Chromosome karyotype analysis of AL patients.

Indexes	AML (*n* = 195)	ALL (*n* = 146)
Normal karyotypes	26 (13.33%)	18 (12.33%)
Complex karyotypes	51 (26.15%)	45 (30.82%)
+10	8 (4.10%)	14 (9.59%)
-20	7 (3.59%)	21 (14.38%)
+8	0 (0.00%)	9 (6.16%)
+4	8 (4.10%)	10 (6.85%)
+14	0 (0.00%)	9 (6.16%)
-7	14 (7.18%)	11 (7.53%)
t(9;22)(q34;q11.2)	5 (2.56%)	5 (3.42%)
t(15;17)(q24;q21)	18 (9.23%)	0 (0.00%)

### 3.8 Correlation analysis between the time from diagnosis to transfer to the ICU and various clinical characteristics

As shown in Table 8, the correlation coefficients between Age (*r* = 0.190), Leukemia type (*r* = −0.109), Heart failure (*r* = −0.176), combine two or more reasons (*r* = 0.078), APACHE II score (*r* = 0.056), GCS scores (*r* = 0.068), MMSE scores (*r* = 0.312), WBC (*r* = −0.268), PLT (*r* = −0.267), LDH (*r* = −0.189), PCT (*r* = 0.275), APTT (*r* = 0.319) and the time from diagnosis to transfer to the ICU in AL patients were relatively high (*p* < 0.05).

**Table 8:** Association between the time from diagnosis to transfer to the ICU and clinical characteristics in AL patients.

Variables	*r*	*P* value
Age (years)	0.190	0.000
Gender (male/female)	0.063	0.098
Leukemia type (AML/ALL)	−0.109	0.039
APACHE II (scores)	0.056	0.029
GCS (scores)	0.068	0.019
MMSE (scores)	0.312	0.000
Reasons for transfer to ICU		
Respiratory failure (Yes, No)	−0.012	0.828
Heart failure (Yes, No)	−0.176	0.001
Liver and kidney failure (Yes, No)	−0.036	0.494
Severe infection (Yes, No)	−0.019	0.720
Clinically uncontrollable coagulation dysfunction (Yes, No)	−0.018	0.732
Shock (Yes, No)	0.020	0.704
ARDS (Yes, No)	−0.022	0.676
Sepsis (Yes, No)	−0.013	0.807
Combine two or more reasons (Yes, No)	0.078	0.012
Laboratory indexes		
WBC (10^9/L)	−0.268	0.000
HGB (g/L)	−0.026	0.492
PLT (10^9/L)	−0.267	0.000
LDH (U/L)	−0.189	0.000
PCT (ng/mL)	0.275	0.000
IL-6 (pg/mL)	−0.045	0.399
PT (s)	−0.015	0.778
INR	0.073	0.177
APTT (s)	0.319	0.000
FIB (g/L)	0.083	0.120
D-dimer (mg/L)	−0.070	0.192

### 3.9 Association between the time from diagnosis to transfer to ICU and aberrant genes in AL patients

As shown in Tables 9, 10, the correlation coefficients between WT1 (*r* = −0.186), FLT3-ITD (*r* = −0.076), TP53 (*r* = 0.013) and the time from diagnosis to transfer to the ICU in AML patients were relatively high (*p* < 0.05). The correlation coefficients between FLT3-ITD (*r* = −0.085), E2A-PBX1 (*r* = −0.162), DNMT3A (*r* = −0.236), HOX11 (*r* = 0.526), RUNX1 (*r* = −0.067) and the time from diagnosis to transfer to the ICU in ALL patients were relatively high (*p* < 0.05).

**Table 9:** Association between the time from diagnosis to transfer to ICU and aberrant genes in AML patients.

Variables	*r*	*P* value
WT1	−0.186	0.002
FLT3-ITD	−0.076	0.014
PML::RARα	−0.376	0.176
TP53	0.013	0.004
NPM1	−0.246	0.432
MLL/AF9	−0.087	0.219
CBFβ-MYH11	−0.145	0.536
TEL-AML1	−0.009	0.781
AML1-ETO	−0.418	0.634
PLZF::RARα	0.066	0.079
STAT5b::RARα	−0.036	0.092

**Table 10:** Association between the time from diagnosis to transfer to ICU and aberrant genes in ALL patients.

Variables	*r*	*P* value
BCR::ABL1	−0.263	0.258
FLT3-ITD	−0.085	0.021
E2A-HLF	−0.016	0.069
E2A-PBX1	−0.162	0.001
SIL-TAL1	0.145	0.189
DNMT3A	−0.236	0.007
EBPA	0.124	0.084
NRAS	−0.685	0.302
TP53	−0.016	0.156
EVI1	0.491	0.254
HOX11	0.526	0.034
RUNX1	−0.067	0.046
TET2	−0.865	0.354
GATA	−0.346	0.089

### 3.10 Risk factor analysis for AL patient mortality in ICU

Through statistical analysis of clinical data from the included AL patients, as shown in the univariate analysis in Table 11, factors such as heart failure, sepsis, CRRT, simultaneous administration of two or more treatments, APACHE II score ≥20, and PCT levels were associated with prognosis.

**Table 11:** Univariate analysis of risk factors for death in AL patients transferred to ICU.

Causes of death and related factors	All patients (*n* = 357)	Outcome during ICU stay		χ^2^ /Z	*P* value
		Survivors (*n* = 42)	Non-survivors (*n* = 315)		
Age (years)	42.15 [14.00–65.00]	46.50 [10.50–63.00]	51.00 [14.00–66.00]	0.839	0.401
Gender (male/female)	216/141	21/21	195/120	0.615	0.141
Leukemia type(AML/ALL)	204/153	26/16	178/137	0.800	0.507
APACHE II scores					
0 ~ 10	25	6 (24.00%)	19 (76.00%)	3.184	0.074
11 ~ 15	42	9 (21.43%)	33 (78.57%)	3.148	0.076
15 ~ 20	98	18 (18.37%)	80 (81.63%)	2.928	0.087
≥ 20	192	11 (5.73%)	181 (94.27%)	5.215	0.022
GCS scores					
< 3	14	4 (28.57%)	10 (71.43%)	3.503	0.061
3 ~ 8	37	8 (21.62%)	29 (78.38%)	2.940	0.086
9 ~ 12	208	35 (16.83%)	178 (83.17%)	2.861	0.091
13 ~ 15	98	18 (18.37%)	80 (81.63%)	2.928	0.087
MMSE scores					
0 ~ 9	11	3 (27.27%)	8 (72.73%)	2.391	0.122
10 ~ 20	29	6 (20.69%)	23 (79.31%)	1.962	0.161
21 ~ 26	174	26 (14.94%)	148 (85.06%)	1.058	0.304
27 ~ 30	143	24 (16.78%)	119 (83.22%)	2.244	0.134
Reasons for transfer to ICU					
Respiratory failure	87	11 (12.64%)	76 (87.36%)	0.896	0.770
Heart failure	27	9 (33.33%)	18 (66.67%)	4.500	0.001
Liver and kidney failure	18	4 (22.22%)	14 (77.78%)	2.263	0.168
Severe infection	48	6 (12,50%)	42 (87.50%)	1.083	0.865
Clinically uncontrollable coagulation dysfunction	35	3 (8.57%)	32 (91.43%)	0.964	0.948
Shock	13	2 (15.38%)	11 (84.62%)	1.382	0.681
ARDS	10	0(0.00%)	10 (100.00%)	0.000	0.999
Sepsis	54	1 (1.85%)	53 (98.15%)	0.121	0.039
Combine two or more reasons	65	5 (7.69%)	60 (92.31%)	0.574	0.265
Treatment measures after being transferred to ICU					
Use vasoactive drugs	56	4 (7.14%)	52 (92.86%)	1.086	0.328
Mechanical ventilation	75	6 (8.00%)	69 (92.00%)	0.594	0.364
Plasmapheresis	6	0 (0.00%)	6 (100.00%)	0.000	0.999
CRRT	42	12 (28.57%)	30 (71.43%)	3.800	<0.001
Hormone shock therapy	76	12 (15.79%)	64 (84.21%)	1.569	0.129
Two or more treatments are given simultaneously	102	5 (4.90%)	97 (95.10%)	0.304	0.027
Laboratory indexes					
WBC	104.81 [0.93–88.70]	24.55 [2.04–90.81]	24.56 [0.90–88.70]	0.573	0.567
HGB	20.59 [45.00–68.00]	58.00 [46.00–70.50]	57.00 [45.00–68.00]	0.702	0.483
PLT	34.29 [5.00–44.00]	15.00 [5.75–53.00]	9.00 [5.00–30.00]	1.621	0.105
LDH	2146.48 [383.00–2125.00]	586 [398–1743.25]	834 [383–2,193]	1.074	0.283
PCT	15.24 [0.69–10.80]	0.86 [0.26–4.30]	2.86 [0.90–18.42]	3.377	0.001
IL-6	1618.76 [23.81–242.24]	100.00 [24.23–234.00]	108.00 [23.39–242.24]	0.447	0.655
PT	20.81 [14.10–20.40]	15.80 [13.95–17.95]	16.65 [14.10–20.60]	1.388	0.165
INR	1.88 [1.21–1.77]	1.38 [1.22–1.57]	1.45 [1.21–1.81]	1.326	0.185
APTT	42.20 [28.20–45.30]	34.30 [28.10–43.40]	35.70 [28.20–45.53]	0.909	0.363
FIB	3.26 [1.30–5.17]	2.42 [1.21–5.26]	2.58 [1.40–5.22]	0.823	0.410
D-dimer	22.02 [3.42–31.42]	11.33 [2.94–27.35]	6.96 [3.42–32.10]	0.201	0.840
Length of stay in ICU (days)	4.13 [1.00–5.00]	3.00 [2.00–5.00]	3.00 [1.00–5.00]	0.427	0.669
Duration of AL disease (months)	5.89 [0.06–8.00]	0.85 [0.13–2.50]	1.00 [0.06–8.00]	0.176	0.861

**Figure 9:**
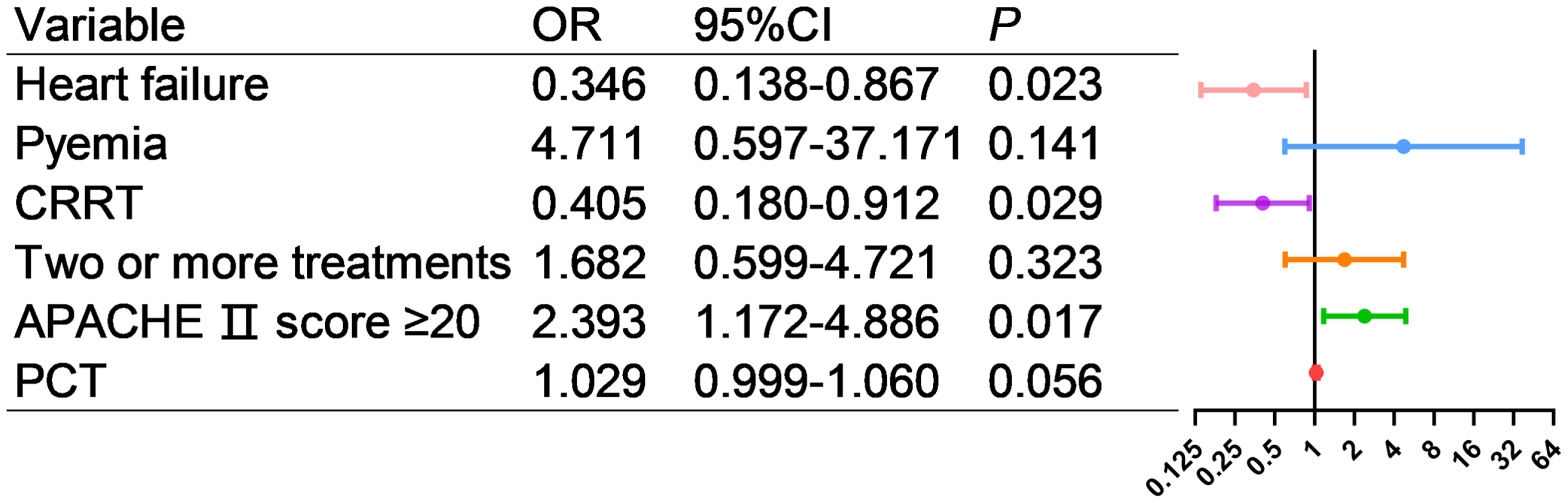
Multivariate logistic regression analysis of risk factors for death in AL patients transferred to ICU.

As shown in Figure 9, factors with *p* < 0.05 in the univariate analysis were included in multivariate analysis, revealing that heart failure, CRRT, and APACHE II score ≥20 were independent risk factors for mortality.

### 3.11 Cox regression analysis

As shown in Table 12, the age, sex, type of leukemia, reason for ICU admission, treatment measures post-admission, APACHE II score, GCS score, MMSE score and other indicators of the 357 cases of AL patients transferred to the ICU were included in the Cox regression model for OS univariate analysis. Results indicated that heart failure, use of vasoactive drugs, and APACHE II score were influencing factors for patient OS.

**Table 12:** Univariate Cox regression analysis of OS in AL patients transferred to ICU.

Clinical features	*n* (%)	χ^2^ value	*P* value
Age (years)		0.001	0.970
≤ 60	243 (68.07)		
> 60	114 (31.93)		
Gender		0.784	0.376
Male	216 (60.50)		
Female	141 (39.50)		
Type of AL		1.333	0.248
AML	204 (57.14)		
ALL	153 (42.86)		
Reason for ICU admission			
Respiratory failure	87 (24.37)	2.734	0.098
Heart failure	27 (7.56)	5.980	0.015
Liver and kidney failure	18 (5.04)	0.119	0.730
Severe infection	48 (13.45)	3.489	0.062
Clinically uncontrollable coagulation dysfunction	35 (9.80)	0.026	0.872
Shock	13 (3.64)	1.204	0.273
ARDS	10 (2.80)	0.720	0.396
Sepsis	54 (15.13)	2.683	0.101
Combine two or more reasons	65 (18.21)	5.664	0.994
Treatments			
Use vasoactive drugs	56 (15.69)	9.975	0.002
Mechanical ventilation	75 (21.01)	0.256	0.613
Plasmapheresis	6 (1.68)	0.388	0.533
CRRT	42 (11.76)	0.039	0.844
Hormone shock therapy	76 (21.29)	0.484	0.487
Two or more treatments are given simultaneously	102 (28.57)	1.042	0.307
APACHE II (score)		4.430	0.035
0~10	25 (7.00)		
11~15	42(11.76)		
15~20	98 (27.45)		
≥ 20	192 (53.78)		
GCS (scores)		2.982	0.147
< 3	14 (3.92)		
3 ~ 8	37 (10.37)		
9 ~ 12	208 (58.26)		
13 ~ 15	98 (27.45)		
MMSE (scores)		0.576	0.452
0 ~ 9	143 (40.06)		
10 ~ 20	29 (8.12)		
21 ~ 26	11 (3.08)		
27 ~ 30	174 (48.74)		

**Figure 10:**
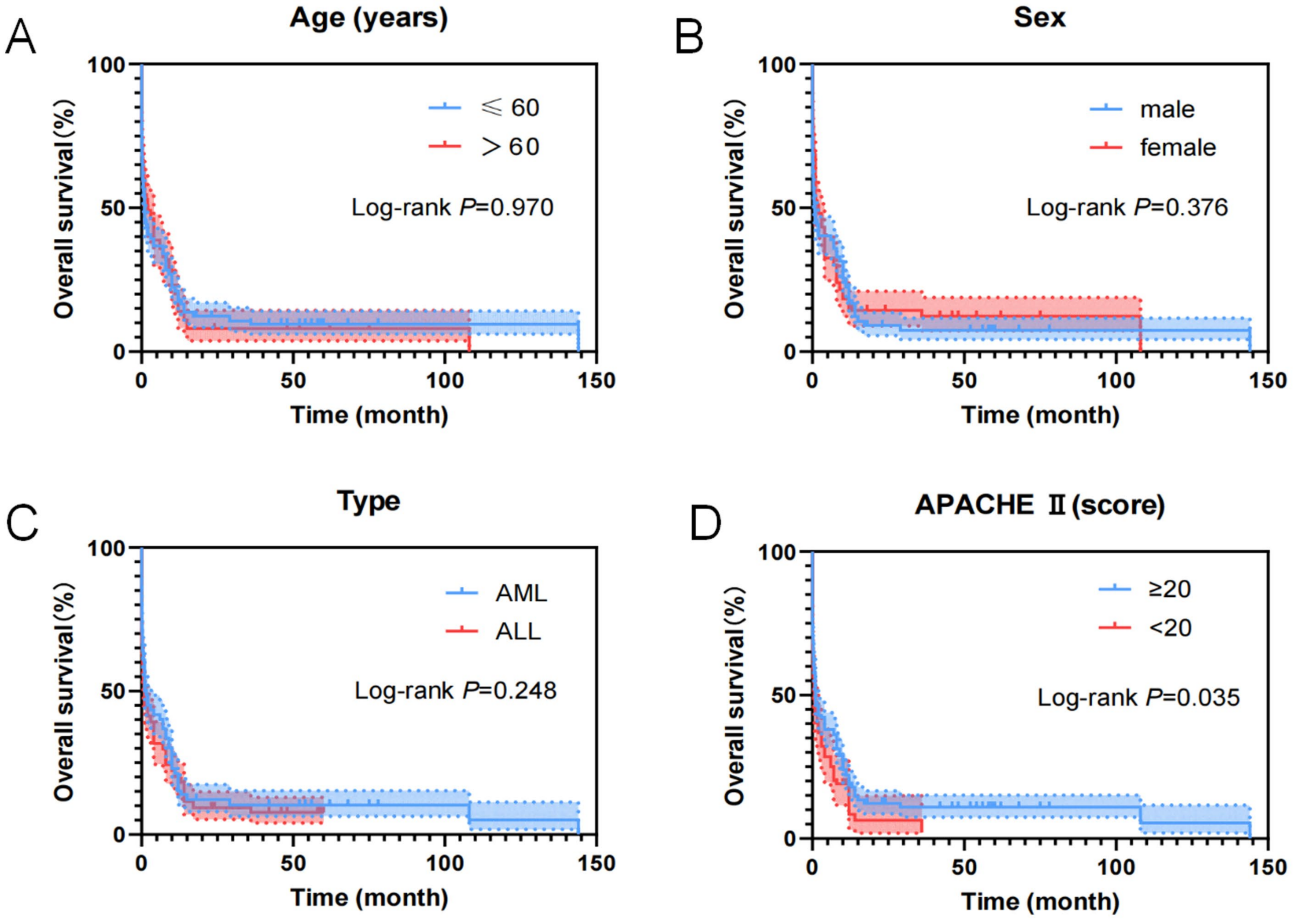
Kaplan–Meier overall survival curves for clinical features of AL patients transferred to ICU. **(A)** The overall survival curves of age of AL patients transferred to ICU. **(B)** The overall survival curves of sex of AL patients transferred to ICU. **(C)** The overall survival curves of disease type of AL patients transferred to ICU. **(D)** The overall survival curves of APACHE II score of AL patients transferred to ICU.

As shown in Figure 10, general clinical data indicators of the 357 cases of AL patients transferred to the ICU were analyzed using the Cox regression model for univariate OS analysis, showing that the APACHE II score was an influential factor for patient OS.

**Figure 11:**
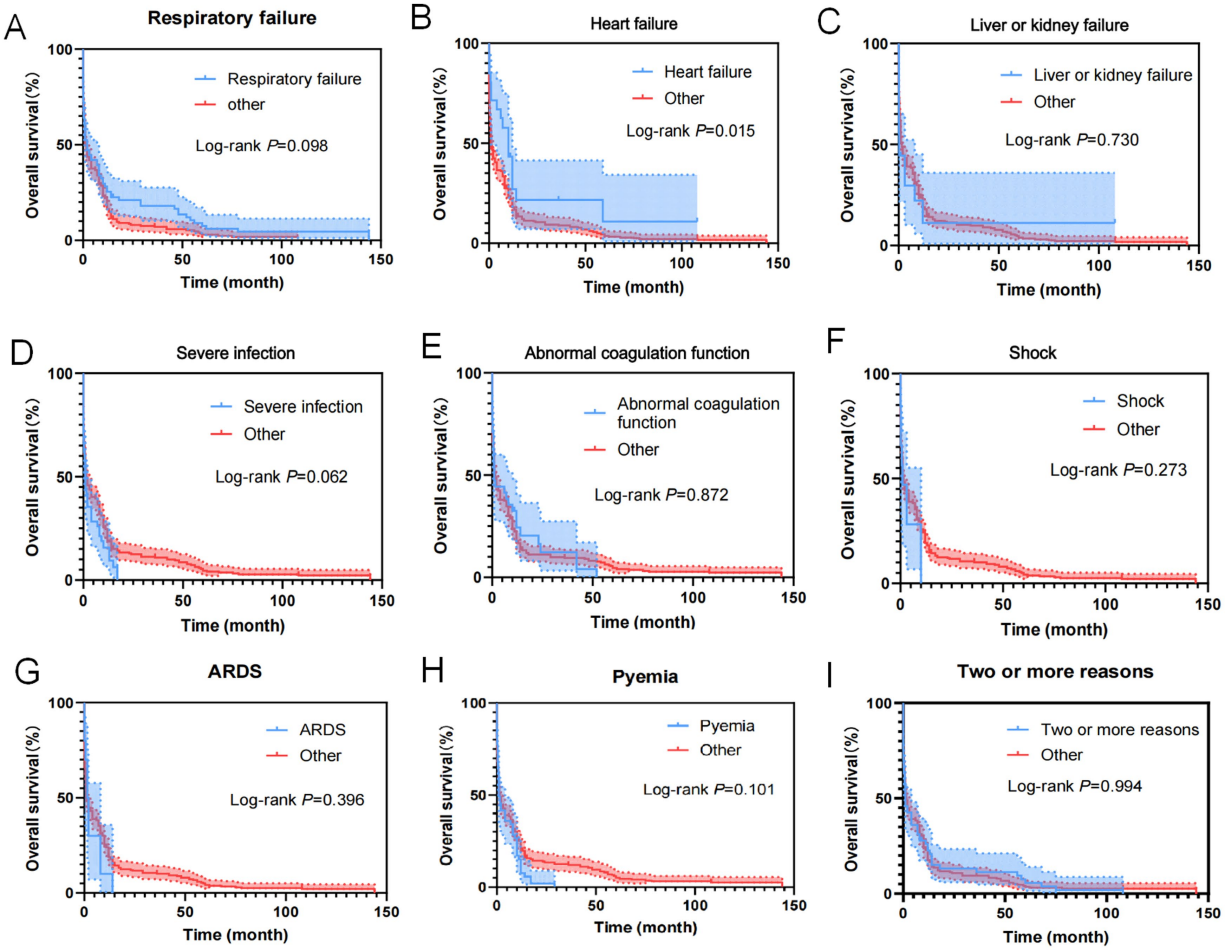
Kaplan–Meier overall survival curves for reasons of AL patients transferred to ICU. **(A)** The overall survival curves of AL patients transferred to ICU because of respiratory failure. **(B)** The overall survival curves of AL patients transferred to ICU because of heart failure. **(C)** The overall survival curves of AL patients transferred to ICU because of liver or kidney failure. **(D)** The overall survival curves of AL patients transferred to ICU because of severe infection. **(E)** The overall survival curves of AL patients transferred to ICU because of abnormal coagulation function. **(F)** The overall survival curves of AL patients transferred to ICU because of shock. **(G)** The overall survival curves of AL patients transferred to ICU because of ARDS. **(H)** The overall survival curves of AL patients transferred to ICU because of pyaemia. **(I)** The overall survival curves of AL patients transferred to ICU because of two or more reasons.

As shown in Figure 11, reasons for ICU admission of the 357 cases of AL patients were analyzed using the Cox regression model for univariate OS analysis, indicating that heart failure was an influential factor for patient OS.

**Figure 12:**
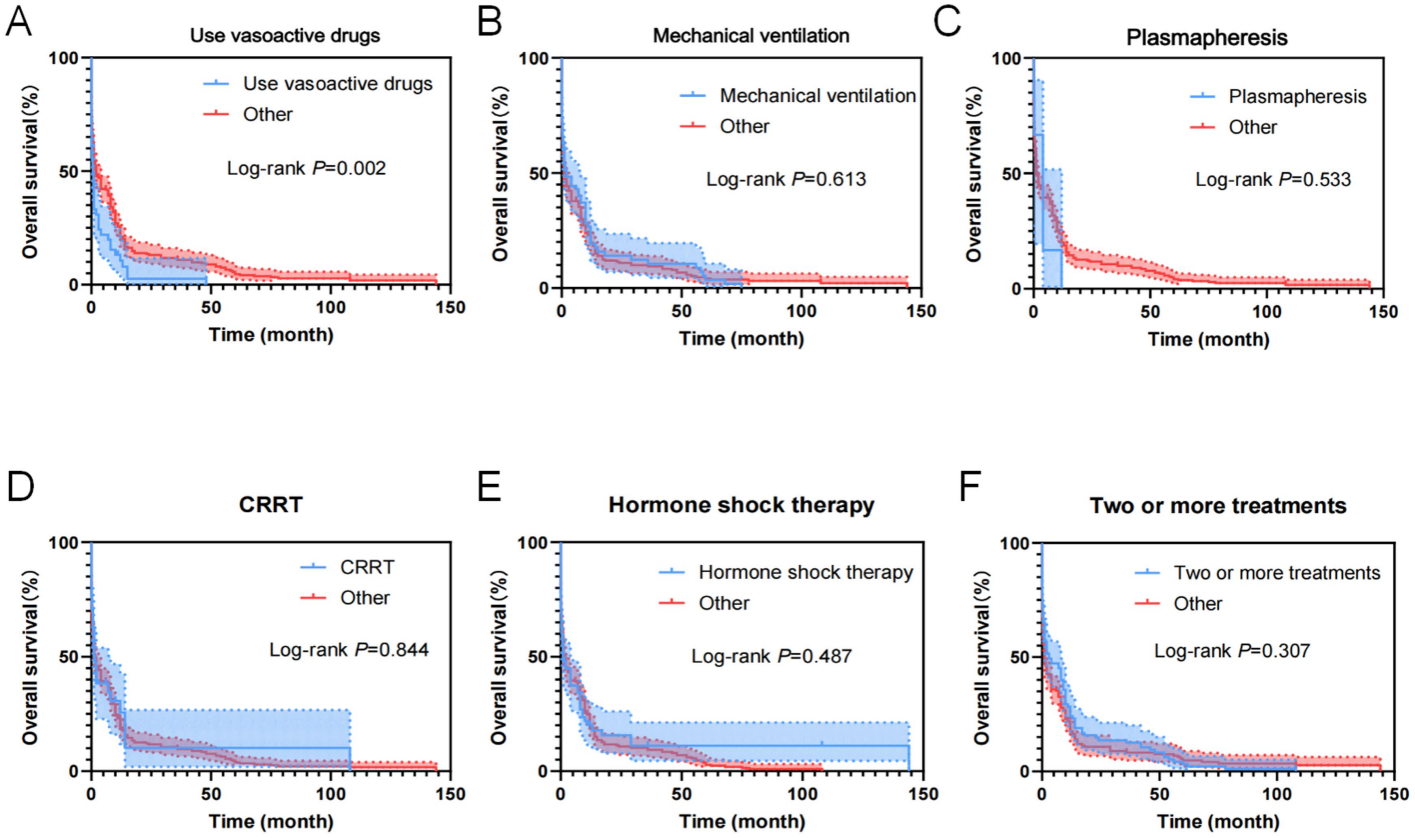
Kaplan–Meier overall survival curves for treatments of AL patients transferred to ICU. **(A)** The overall survival curves of AL patients transferred to ICU used Vasoactive drugs. **(B)** The overall survival curves of AL patients transferred to ICU used mechanical ventilation. **(C)** The overall survival curves of AL patients transferred to ICU used plasmapheresis. **(D)** The overall survival curves of AL patients transferred to ICU used CRRT. **(E)** The overall survival curves of AL patients transferred to ICU used hormone shock therapy. **(F)** The overall survival curves of AL patients transferred to ICU used two or more treatments.

As shown in Figure 12, treatment measures of the 357 cases of AL patients transferred to the ICU were analyzed using the Cox regression model for univariate OS analysis, demonstrating that the use of vasoactive drugs was an influential factor for patient OS.

As shown in Table 13, variables with *p* < 0.05 in the univariate analysis were included in the multivariate analysis model, revealing that the use of vasoactive drugs was an independent risk factor for patient OS.

**Table 13:** Multi-factor Cox regression analysis of OS in AL patients transferred to ICU.

Clinical features	OR	95%CI	*P* value
APACHE II (score)	0.822	0.633–1.068	0.142
Heart failure	0.973	0.685–1.383	0.881
Use vasoactive drugs	1.414	1.115–1.795	0.004

## 4 Discussion

AL is a life-threatening hematological malignancy characterized by the clonal proliferation of poorly differentiated hematopoietic progenitor cells. Specifically, myeloblasts are involved in AML, while lymphoblastic cells dominate in ALL. This leads to bone marrow infiltration. Advances in molecular biology, cytogenetics, and immunology have improved survival rates and quality of life for patients with AL to some extent. However, AL remains a highly aggressive malignant hematological tumor with severe clinical manifestations. General hematology wards often lack sufficient medical staff to provide one-to-one care for AL patients, resulting in delayed treatment and irreversible damage due to rapid changes in their condition. With increasing incidence and risk groups, as well as declining mortality over time, AL patients are expected to require higher utilization of ICU resources in the future.

Routine hematological indicators in this study revealed severe leukocytosis or reduction, thrombocytopenia, and anemia in most patients. Cytogenetic and molecular abnormalities are prevalent in AL patients, with high-risk genetic abnormalities (e.g., complex karyotypes, TP53 mutations) strongly linked to poor prognosis. Complex cytogenetic abnormalities may enhance resistance of leukemia cells to chemotherapy drugs. This study revealed that, irrespective of whether it was AML or ALL, patients with complex karyotypes constituted the largest proportion. Therefore, intensive treatment strategies such as allogeneic hematopoietic stem cell transplantation (allo-HSCT) should be considered early for patients with complex cytogenetic abnormalities.

As is known to all, molecular markers (e.g., FLT3-ITD, NPM1 mutation) are valuable for disease classification, prognosis assessment, and targeted therapy. Our study identified that FLT3-ITD, PML-RARα, TEL-AML1, and AML1-ETO are associated with the prognosis of AML patients. WT1, FLT3-ITD, and TP53 significantly influence the time from diagnosis to transfer to the ICU in AML patients. The AML mutation spectrum discovered in this study is consistent with previous reports (18, 19), including reproducible alterations in FLT3, WT1, NRAS, KRAS, and ARID1A genes, as well as other rare mutant genes in AML (DNMT3A, TET2, ASXL1, and IDH1/2). WT1, FLT3-ITD, and TP53 can serve as early warning indicators for AML patients requiring ICU transfer or experiencing short-term mortality. For ALL patients, common mutations include NRAS, KRAS, FLT3, JAK3, GATA3, RUNX1, and EZH2. In addition to these common mutations, ALL patients exhibit a higher incidence of DNA methylation mutations (DNMT3A, IDH1, IDH2, TET2, TET3, and WT1) (20, 21). And our study demonstrates that BCR-ABL1, FLT3-ITD, E2A-PBX1, DNMT3A, NRAS, EVI1, HOX11, RUNX1, and GATA are related to the prognosis of ALL patients. FLT3-ITD, E2A-PBX1, DNMT3A, HOX11, and RUNX1 significantly affect the time from diagnosis to ICU transfer in ALL patients, findings which align with literature reports. These genetic markers (FLT3-ITD, E2A-PBX1, DNMT3A, HOX11, and RUNX1) are anticipated to become early warning indicators for ICU transfer or short-term mortality in ALL patients, providing critical insights into poor prognosis and imminent death.

Approximately 30,000 new cases of AL are diagnosed annually in the United States. Despite improvements in survival due to advancements in technology and drugs, many AL patients still experience acute complications requiring ICU admission for further treatment. ICU admission typically indicates a complex clinical condition with poor prognosis. Data demonstrate that AL patients requiring ICU admission exhibit high mortality rates (22, 23). Studies confirm that although the relative risk of death decreases over time for ICU-admitted patients, the risk remains significantly higher than in the general population after 3 to 5 years (24). Early critical illness onset generally occurs within 4 weeks post-diagnosis and includes hyperleukocytosis or leukostasis, acute respiratory failure or respiratory distress syndrome, tumor lysis syndrome (TLS), disseminated intravascular coagulation (DIC), and severe bleeding. Timely recognition and treatment of these conditions are crucial to prevent mortality. Hematology clinicians must update their understanding of critical AL, identify ICU admission risk factors, and monitor high-risk patients early to improve outcomes. A systematic retrospective analysis of 357 cases of AL patients in our study revealed that acute respiratory failure, sepsis, and severe infection were the primary reasons for ICU admission, while heart failure, CRRT, and APACHE II scores ≥20 were independent prognostic factors.

Leukostasis arises from an abnormal increase in leukocyte count, leading to elevated blood viscosity, reduced blood flow, and impaired tissue perfusion, which manifest as various clinical symptoms. The critical white blood cell threshold varies depending on the type of leukemia; even below this threshold, complications can occur. Organ hypoxia is hypothesized to be the main pathophysiological change in leukostasis, though the exact mechanism of tissue hypoxia remains unclear (25). Clinical symptoms of leukostasis primarily involve central nervous system (about 40%) and lung (about 30%) involvement, including dyspnea, hypoxia, visual changes, headache, dizziness, gait instability, confusion, lethargy, intracranial hemorrhage, and brain herniation. Leukapheresis remains the most effective treatment for leukostasis, removing white blood cells from peripheral circulation. Prophylactic use is recommended when the white blood cell count exceeds 100 × 10^9^/L in AML patients and 200 × 10^9^/L in ALL patients. However, there is no consensus on the impact of leukapheresis on short-term and long-term patient outcomes (26, 27).

Acute respiratory failure was a common cause of ICU admission for AL patients in this study. Contributing factors include lung infections, alveolar bleeding, chemotherapy-related pulmonary toxicity, and leukemic pulmonary infiltration. Chemotherapy-associated pulmonary toxicity is frequently observed with drugs such as methotrexate, cyclophosphamide, and cytarabine, manifesting as interstitial pneumonia, pulmonary fibrosis, or acute respiratory distress syndrome (ARDS). Pulmonary toxicity typically occurs within days to weeks after chemotherapy initiation and may correlate with cumulative drug doses (28). Leukemic pulmonary infiltration is more prevalent in AML patients, particularly those with hyperleukocytosis (29). In cases of acute respiratory failure, airway obstructions should be promptly removed to ensure smooth airflow. Intubation by the anesthesia department or tracheotomy by the ENT department may be necessary to establish an artificial airway. Simultaneously, arterial blood gas analysis should guide appropriate oxygen therapy. Mechanical ventilation is indicated for patients with altered consciousness, progressive hypoxemia (PaO₂ < 50 mmHg), or hypercapnia (PaCO₂ > 70 mmHg), with non-invasive ventilation offering clinical benefits (30, 31).

Severe infection is the most frequent reason for ICU admission in AL patients, neutropenia and immune dysfunction predispose AL patients to bacterial, fungal, or viral infections, especially in neutropenic patients who are at higher risk for serious infections such as sepsis and pneumonia. Fungal infections, particularly invasive Aspergillus infections, are common in AL patients and associated with poor prognosis (32), necessitating advanced and complex antimicrobial regimens. Broad-spectrum antibiotics targeting gram-negative bacteria, including anti-pseudomonas *β*-lactam drugs, carbapenems, or piperacillin-tazobactam, along with appropriate antifungal prophylaxis, are commonly used. Cytomegalovirus and Pneumocystis infections are also significant contributors to severe infections in AL patients, findings consistent with international research (33–35). The several medical centers participating in our study adhered to the principles of individualized treatment, multidisciplinary collaboration, and dynamic adjustment in their antibacterial strategies, antifungal prophylaxis, and supportive care protocols for acute leukemia patients transferred to the intensive care unit. Undoubtedly, early empirical antibacterial therapy, appropriate antifungal prophylaxis, and comprehensive supportive care are critical factors in reducing infection-related mortality. Furthermore, effective infection control measures and antimicrobial stewardship play essential roles in limiting the spread of drug-resistant organisms and improving patient outcomes.

Uncontrollable coagulation dysfunction is a key predictor of poor prognosis in AL patients. Leukemia cells infiltrate and destroy vascular endothelial cells, disrupting coagulation and anticoagulation balance, leading to microthrombosis and bleeding tendencies. DIC manifests as skin petechiae, ecchymosis, mucosal bleeding, and gastrointestinal bleeding. Severe cases may result in multiple organ failure. Up to 32% of non-APL-AML patients exhibit significant DIC abnormalities (36). Early hypercoagulable DIC may present without symptoms or mild symptoms but can progress to thromboembolism and shock. Extensive and uncontrollable multi-site bleeding occurs during consumptive hypocoagulability and secondary hyperfibrinolysis (37). Mortality increases significantly in patients with severe bleeding complications such as intracranial hemorrhage and gastrointestinal bleeding. Anticoagulation therapy is the primary treatment, supplemented by coagulation factor replacement, mainly fresh frozen plasma infusion, and low molecular weight heparin administration (38, 39).

APL is distinguished by pronounced hypofibrinogenemia and high early mortality due to coagulation and bleeding complications. The prognosis of patients treated with all-trans retinoic acid (ATRA) has significantly improved. Differentiation syndrome is another fatal complication during remission therapy induced by retinoic acid or arsenic trioxide. Originally termed retinoic acid syndrome, its mechanism remains incompletely understood. Current studies suggest induction therapy increases cytokine (IL-1, IL-6, TNF-*α*) and adhesion molecules (CD116, CDw65, VLA-4, CD11a/CD54) secretion, promoting APL cell migration to the lungs and causing clinical symptoms such as fever, weight gain, musculoskeletal pain, respiratory distress, pulmonary interstitial infiltration, pleural effusion, pericardial effusion, skin edema, hypotension, acute renal failure, and death. It typically occurs within 10 to 12 days after ATRA initiation, with early identification and hormone therapy being critical (40, 41).

Heart failure in AL patients significantly increases the risk of poor prognosis. According to Kang Y et al.’s study, the incidence of heart failure in AL patients is higher than in the general population due to cardiotoxicity from chemotherapy drugs (e.g., anthracyclines), infective endocarditis, and anemia (42, 43). Heart failure-induced cardiac output reduction exacerbates tissue hypoxia, affects chemotherapy drug metabolism and excretion, and creates a vicious cycle (44).

Renal impairment in AL patients is primarily related to TLS, sepsis, and nephrotoxic drug use. CRRT application corrects electrolyte disturbances and maintains internal stability but reflects severe multi-organ dysfunction (45, 46). TLS is a life-threatening complication during induction chemotherapy, commonly seen in high-white ALL or AML and high-tumor load non-Hodgkin lymphoma. Since AML accounts for about 80% of adult cases, AML-associated TLS with increased white blood cells is clinically more common. TLS features hyperuricemia, hyperkalemia, hyperphosphatemia, hypocalcemia, and acute renal failure, occurring within 3 days before and 7 days after chemotherapy initiation. Treatment includes intravenous fluids, electrolyte correction, labrylase use, diuretics, and renal replacement therapy as needed (47, 48).

The APACHE II score is a critical tool for evaluating ICU patient severity and prognosis. By comprehensively assessing physiological indicators, age, and chronic health status, the APACHE II score accurately predicts mortality risk. Higher APACHE II scores correlate with significantly increased mortality rates, consistent with international research findings (49–51). Combining APACHE II scores with other prognostic factors (e.g., age, comorbidities, molecular biological characteristics) enhances prediction accuracy. A cohort study of 167 AL patients confirmed APACHE II score as an independent prognostic factor for ICU-admitted AL patients (52). Vasoactive drug use was identified as an independent risk factor for OS in patients. Patients requiring vasoactive drugs often suffer from septic shock, cardiac insufficiency, or other forms of circulatory failure. Due to low immune function, AL patients are prone to severe infections leading to sepsis and septic shock. Multiple studies associate vasoactive drug requirements (e.g., norepinephrine) with poor prognosis (6, 53, 54).

Notably, in our study, heart failure, CRRT, and APACHE II scores ≥20 were identified as independent risk factors affecting prognosis. Clinicians should focus on evaluating heart and kidney function and disease severity in AL patients with these risk factors, implementing timely interventions such as infection prevention and organ support to improve outcomes. These risk factors also serve as important references for clinical decision-making, aiding in disease severity and prognosis evaluation. Multidisciplinary collaboration involving hematology, ICU, and infectious disease specialists is essential for comprehensive AL patient management. Novel therapies (e.g., immunotherapy, targeted therapy) show promise in improving outcomes for high-risk AL patients. For instance, CAR-T cell therapy demonstrates efficacy in relapsed/refractory AL patients (55, 56).

Currently, domestic and international studies rarely address early warning indicators for AL patients requiring ICU transfer. This study reveals that age, leukemia type, heart failure, presence of two or more comorbidities, APACHE II score, WBC, PLT, LDH, PCT, and APTT significantly impact the time from diagnosis to ICU transfer in AL patients. Clinical practitioners should prioritize monitoring these indicators, as they hold significant prognostic value and guide the identification of severe cases in the short term.

This study did not only address the effects of novel treatments on prognosis, highlighting a direction for future research. Future studies could explore the efficacy and safety of novel therapeutic approaches (e.g., CAR-T cell therapy, targeted therapy) in ICU patients and how they integrate with traditional strategies to optimize overall treatment outcomes in AL patients. Additionally, precision medicine development will enable personalized treatment strategies based on molecular biological characteristics, improving patient outcomes and quality of life. Honestly, it is acknowledged that this study may have some limitations related to study design, and analytical methods. To address these limitations, several methodological measures were implemented, including comprehensive reporting of baseline demographics and treatment protocols; application of multivariate regression models to adjust for potential confounders; performance of subgroup analyses to assess heterogeneity; cautious interpretation of results without implying causal relationships; and recommendations for future prospective, large-scale, multicenter studies to confirm the findings.

## 5 Conclusion

AL patients transferred to the ICU exhibit complex conditions and poor prognosis. Acute respiratory failure, sepsis, and severe infection are the primary causes of hospitalization, while heart failure, CRRT, and APACHE II scores ≥20 are independent risk factors for mortality. Vasoactive drug use is an independent OS risk factor. Early identification of high-risk patients, multidisciplinary collaboration, and incorporation of novel therapies may improve patient outcomes. Further studies are needed to optimize ICU management strategies for AL patients.

## Data Availability

The original contributions presented in the study are included in the article/supplementary material, further inquiries can be directed to the corresponding authors.
